# Design and Synthesis of New Benzothiazole Compounds as Selective *h*MAO-B Inhibitors

**DOI:** 10.3390/molecules22122187

**Published:** 2017-12-09

**Authors:** Sinem Ilgın, Derya Osmaniye, Serkan Levent, Begüm Nurpelin Sağlık, Ulviye Acar Çevik, Betül Kaya Çavuşoğlu, Yusuf Özkay, Zafer Asım Kaplancıklı

**Affiliations:** 1 Department of Pharmaceutical Toxicology, Faculty of Pharmacy, Anadolu Universty, 26470, Eskişehir, Turkey; silgin@anadolu.edu.tr; 2 Department of Pharmaceutical Chemistry, Faculty of Pharmacy, Anadolu Universty, 26470, Eskişehir, Turkey; dosmaniye@anadolu.edu.tr (D.O.); serkanlevent@anadolu.edu.tr (S.L.); bnsaglik@anadolu.edu.tr (B.N.S.); uacar@anadolu.edu.tr (U.A.Ç.); betulkaya@anadolu.edu.tr (B.K.Ç.); zakaplan@anadolu.edu.tr (Z.A.K.); 3 Doping and Narcotic Compounds Analysis Laboratory, Faculty of Pharmacy, Anadolu Universty, 26470, Eskişehir, Turkey

**Keywords:** benzothiazole, hydrazone, MAO enzyme inhibition, docking study, cytotoxicity

## Abstract

In the current work a new class of novel benzothiazole-hydrazone derivatives was designed and synthesized as *h*MAO-B inhibitors. Structures of the obtained compounds (**3a**–**3j**) were characterized by IR, ^1^H-NMR, ^13^C-NMR, and HRMS spectroscopic methods. The inhibitory activity of compounds (**3a**–**3j**) against *h*MAO-A and *h*MAO-B enzymes was evaluated by using an in vitro fluorometric method. According to activity results, some of the synthesized compounds displayed selective and significant *h*MAO-B enzyme inhibitor activity. Compound **3e** was the most active derivative in the series with an IC_50_ value of 0.060 µM. Furthermore, cytotoxicity of compound **3e** was investigated and found to be non-cytotoxic. Absorption, distribution, metabolism, and excretion (ADME) and blood-brain barrier (BBB) permeability predictions were performed for all compounds. It was determined that these compounds may have a good pharmacokinetic profiles. Bınding modes between the most active compound **3e** and the *h*MAO-B enzyme were analyzed by docking studies. It was observed that there is a strong interaction between compound **3e** and enzyme active site.

## 1. Introduction

Monoamine oxidase (MAO) is an important flavoenzyme existing in the outer mitochondrial membrane of neuronal, glial, and many other cells, and is responsible for the oxidative deamination of amines in the brain, as well as peripheral tissues to regulate their level. MAO exists in two isoforms: namely, MAO-A and MAO-B, which have been identified based on their amino acid sequences, three-dimensional structure, substrate preference, and inhibitor selectivity [1,2]. Monoamine oxidase enzyme inhibitors (MAOIs), in general, have inhibitory activity against both of MAO-A and MAO-B [3]. However, researchers have focused to develop a selective MAOI to avoid food–drug and drug–drug interactions of non-selective MAOIs, as well as their side effects [4,5]. Selective inhibition of MAO-A, which specifically metabolizes serotonin, norepinephrine, and tyramine is effective in the depression treatment, while selective inhibition of MAO-B, which specifically degrades dopamine, is effective in the treatment of Parkinson’s disease (PD) [5,6]. It is known that PD is a neurodegenerative disorder characterized by progressive loss of dopaminergic neurons in the substantia nigra pars compacta [7,8]. Therefore, in the treatment of PD, it has been targeted to increase dopaminergic activity through dopamine supplementation, decrease dopamine breakdown, or activate dopaminergic receptors [9,10]. MAO-B inhibitors are useful in the treatment of the early stages of PD and, later, as an adjunct to other drug therapies [11,12]. MAO-B inhibitors have a good safety profile. They also possess an effect in the improvement of motor symptoms and, thus, delay the need for levodopa in the treatment of PD. Furthermore, studies have indicated that MAO-B inhibitors have a neuroprotective effect against neurodegeneration [13]. Therefore, the development of novel, selective, and reversible MAO-B inhibitors with fewer side effects is still necessary.

In terms of crystal structure, the *h*MAO-B enzyme possesses two cavities connected by the amino acid ILE199. The entrance cavity has a volume of 290 Å^3^ and, possesses a hydrophobic character. The second cavity, with a volume of 390 Å^3^, contains the substrate binding site in which coenzyme FAD is located. Crystallographic data of the substrate cavity has displayed that the amino acid side chains, coating the cavity, are very hydrophobic and favorable to interact with an amine moiety. The FAD and two closely-parallel tyrosyl residues (398 and 435) constitute an ‘aromatic cage’ [14,15]. 

**Figure 1:**
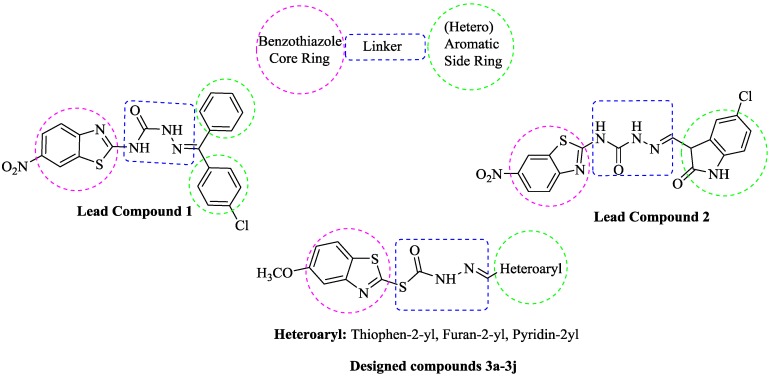
Examples of structures displaying MAO-B inhibitory activity and the designed compounds (**3a–3j**).

The previous studies have shown that benzothiazole derivatives have potent and selective inhibitory activity against the MAO-B enzyme [16,17,18,19]. In these studies, a similar design strategy of target compounds has been followed. Synthesized compounds have been designed to possess three essential parts: (i) a 2-mercapto- or amino-substituted benzothiazole core; (ii) an aromatic or heteroaromatic ring sytem; and (iii) a linker, bearing H-acceptor and/or H-donor nitrogens, between the benzothiazole core and (hetero)aromatic ring system (Figure 1). Docking analyses have revealed that the benzothiazole core binds within the substrate cavity space, the heteroaryl residue of the molecules is located in the entrance cavity space of MAO-B enzyme, and nitrogen atoms of the linker are stabilized by hydrogen bond interactions with amino acid residues of the enzyme active site [16,18]. In addition to benzothiazole derivatives, hydrazine-based compounds, which bear an azomethine -NHN=CH- functional group, have displayed a selective inhibitory activity against MAO-B [18,20,21]. The C=N double bond of hydrazone and the terminal nitrogen atom significantly influence the physical and chemical properties. The C-atom in hydrazone has both electrophilic and nucleophilic properties [22]. Due to MAO-B inhibitory potency and the physicochemical character of the hydrazone moiety, it has been preferred as a linker between the benzothiazole core and the (hetero)aromatic ring system in the design of target compounds as outlined in Figure 1.

In light of the above information, new benzothiazole derivatives consisting of a hydrazone moiety were synthesized as novel, selective, and potent MAO-B inhibitory agents. Additionally, we aimed to determine the in vitro cytotoxic effect of compounds in case they had significant enzyme inhibitory activity.

## 2. Results and Discussion

### 2.1. Chemistry

**Scheme 1:**
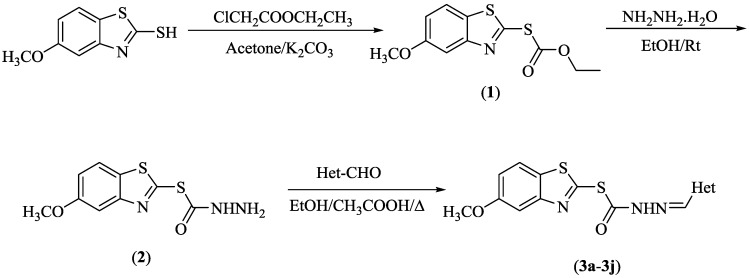
Synthesis pathway of target compounds.

The compounds **3a**–**3j** were synthesized as summarized in Scheme 1. Initially, *O*-ethyl *S*-(5-methoxybenzothiazol-2-yl) (**1**) carbonothioate was prepared by a reaction of 2-mercapto-5-methoxybenzothiazole and ethyl 2-chloroacetate in the presence of potassium carbonate. Then, the reaction of compound **1** and excess of hydrazine hydrate gave *S*-(5-methoxybenzothiazol-2-yl) hydrazinecarbothioate (**2**). In the last step, compounds **3a**–**3j** were prepared via the reaction of compound **2** and an appropriate heterocyclic aldehyde. The structure of the synthesized compounds were elucidated by IR, ^1^H-NMR, ^13^C-NMR, and HRMS analysis. In the IR spectra, the N-H bond of hydrazide was observed over 3061 cm^−1^. Carbonyl and imine groups had bands at 1654–1699 cm^−1^ and 1307–1392 cm^−1^, respectively. In the ^1^H-NMR spectra, protons of methoxy and methylene, neighboring to carbonyl, gave singlet peaks at 3.81–3.82 ppm and 4.55–4.70 ppm, respectively. The benzimidazole H_5_ proton gave a doublet peak at 6.99 ppm with coupling constant values of 8.8 Hz and 2.5 Hz. Benzimidazole H_4_ and H_6_ protons were observed at 7.37–7.42 ppm and 7.86–7.87 ppm. A proton of methine (CH) in the hydrazide moiety was recorded as a singlet peak at 7.87–8.29 ppm. A proton of nitrogen in the same group had a singlet peak at about 11.40 ppm. Other aromatic peaks were observed between 6.09 ppm and 8.88 ppm. In the ^13^C-NMR spectra, aliphatic carbons were observed between 14.01 ppm and 56.01 ppm, while aromatic ones were recorded between 104.96 ppm and 169.23 ppm. In the HRMS spectra, all masses were matched well with the expected M + H values. 

### 2.2. Enzymatic Studies

#### 2.2.1. MAO-A and MAO-B Inhibition Assay

The in vitro fluorometric enzymatic assay, allowing to sensitively detect monoamine oxidase activity, was applied to investigate *h*MAO-A and *h*MAO-B inhibitory potential of compounds **3a**–**3j**. The method was performed in two steps, as previously described [21]. In the first step, compounds **3a**–**3j** were screened at 10^−3^ and 10^−4^ M concentrations to determine the compounds that have more than 50% inhibition potency. Table 1 presents the *h*MAO-A and *h*MAO-B inhibitory activity of compounds **3a**–**3j.** In the second step, selected compounds were used at concentrations of 10^−5^–10^−9^ M to calculate their IC_50_ values (Table 2).

As seen in the Table 1, all compounds showed low inhibitory potency against *h*MAO-A, whereas compounds **3a**, **3e**, **3f**, and **3h** showed more than 50% inhibition against *h*MAO-B and, thus, they were selected for the second step, in which IC_50_ values of 15.450, 0.060, 0.963, and 0.075 μM were recorded, respectively. Selegiline, a standard drug against *h*MAO-B, displayed an IC_50_ of 0.044 μM (Table 2). Enzymatic assay revealed that, synthesized compounds had a selectivity towards *h*MAO-B, as expected. It can be concluded that compound **3e** is the most active compound with a significant IC_50_ of 0.060 μM against *h*MAO-B and, thus, it has been subjected to enzyme kinetic and cytotoxicity assays.

#### 2.2.2. Enzyme Kinetic Studies

The mechanism of *h*MAO-B inhibition was investigated by enzyme kinetic studies. Lineweaver-Burk graphs were used to estimate the type of inhibition. Data were analyzed by recording substrate-velocity curves in the various concentrations (IC_50_/2, IC_50_ and 2 × IC_50_) of the most active compound, **3e**. The velocity measurements were performed by using different substrate concentrations (20 µM–0.625 µM). The K_i_ (intercept on the x-axis) value of compound **3e** was determined from the secondary plot of the 1/V versus varying concentrations.

**Figure 2:**
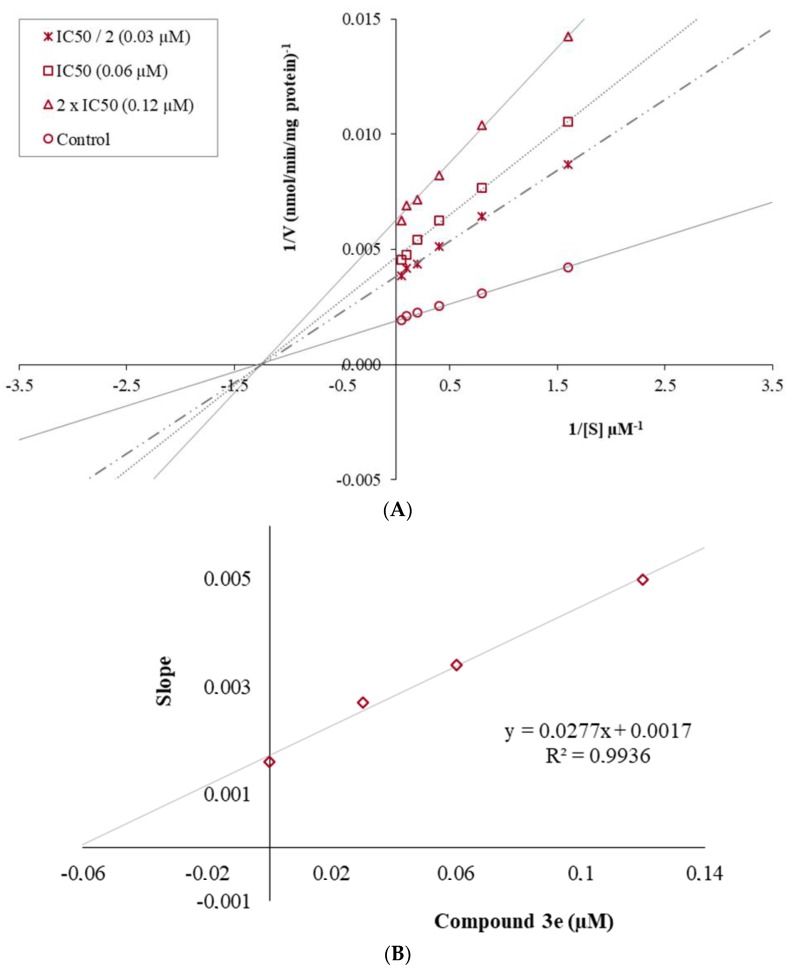
(**A**) Lineweaver–Burk plots for the inhibition of *h*MAO B by compound **3e**. [S], substrate concentration (μM); V, reaction velocity (nmol/min/mg protein). Inhibitor concentrations are shown at the left. V_max_ values from 2 × IC_50_ to Control; 158.730, 212.766, 263.158, and 526.316 (nmol/min/mg protein). K_m_ value of the non-competitive inhibition; 0.767±0.031 (µM). (**B**) Secondary plot for the calculation of the steady-state inhibition constant (K_i_) of compound **3e**. K_i_ was calculated as 0.061 μM.

The graphical analysis of inhibition type for compound **3e** is displayed in Figure 2. The Lineweaver-Burk graphics present the inhibition type as a mixed-type, competitive, uncompetitive, or non-competitive. In the mix-typed inhibition, the control and inhibitor lines cross neither x- nor y-axis at the same point. Competitive inhibitors possess the same intercept on y-axis, but there are diverse slopes and intercepts on x-axis between the two data sets. There is no crosses between the lines in uncompetitive-type inhibition. Non-competitive inhibition has plots with the same intercept on the x-axis, but there are different slopes and intercepts on the y-axis, as seen in the Figure 2. Thus, it can be declared that the mechanism of *h*MAO-B inhibition of **3e** is non-competitive, clarifying that the inhibitor can bind to either free enzyme or enzyme–substrate complex. The K_i_ value for compound **3e** was calculated as 0.061 µM for the inhibition of *h*MAO-B.

### 2.3. Cytotoxicity Test

The healthy NIH/3T3 mouse embryonic fibroblast cell line (ATCC CRL1658), proposed by ISO (10993-5, 2009) for preliminary cytotoxicity screening of drug candidates, was used to evaluate the cytotoxicity of compound **3e** [23]. The IC_50_ value of the compound **3e** is represented in Table 3. It was determined that IC_50_ (19.002 µM) of compound **3e** against NIH/3T3 is ca. 300-fold higher than its IC_50_ (0.06 µM) against *h*MAO-B enzyme. This finding indicates that compound **3e** is not cytotoxic at its effective concentration.

### 2.4. Prediction of ADME Parameters and BBB Permeability 

As an important drug discovery approach it will be beneficial to evaluate the pharmacokinetic profiles of drug candidates during the early development phases. In recent years, by the help of combinatorial chemistry number of drug candidates, for which early data on absorption, distribution, metabolism, and excretion (ADME) are needed, has significantly increased [24]. Thus, predictions of ADME properties of the obtained compounds (**3a**–**3j**) were calculated using online Molinspiration chemical property software [25]. This program is based on to evaluate Lipinski’s rule of five, which predicts the ADME properties of drug like compounds, and is very important to optimize a biologically-active compound. According to Lipinski’s rule, an orally-active drug should not possess more than one violation. The theoretical calculations of ADME parameters (topological polar surface area (TPSA), molecular volume (MV), number of hydrogen acceptors (nOHNH), number of hydrogen donors (nON), partition coefficient (log P), and molecular weight (MW)) are accessible in Table 4 along with the violations of Lipinski’s rule. In regard to these data, the obtained compounds (**3a**–**3j**) suited Lipinski’s rule by displaying no violation. As a result, it can be suggested that the synthesized compounds may have good pharmacokinetic profiles, increasing their pharmacological significance. 

It is known that drugs that specifically target the central nervous system (CNS) have to pass the blood-brain barrier (BBB). Thus, penetration of the BBB is very important for CNS drug candidates, and should be clarified early in drug discovery studies [26]. Therefore, BBB permeability of the synthesized compounds (**3a**–**3j**) was calculated by a CBLigand-BBB prediction server [27]. This online predictor applies two different algorithms, AdaBoost and Support Vector Machine (SVM), combining with four different fingerprints, employed to predict if a compound can pass (+) or cannot pass (−) the BBB. Predictor scores are higher than 0 if a compound can pass the BBB. Calculations for all compounds shwing BBB (+) are shown in Table 4, which are essential for MAO inhibitors to display their biological activity.

### 2.5. Molecular Docking Studies

**Figure 3:**
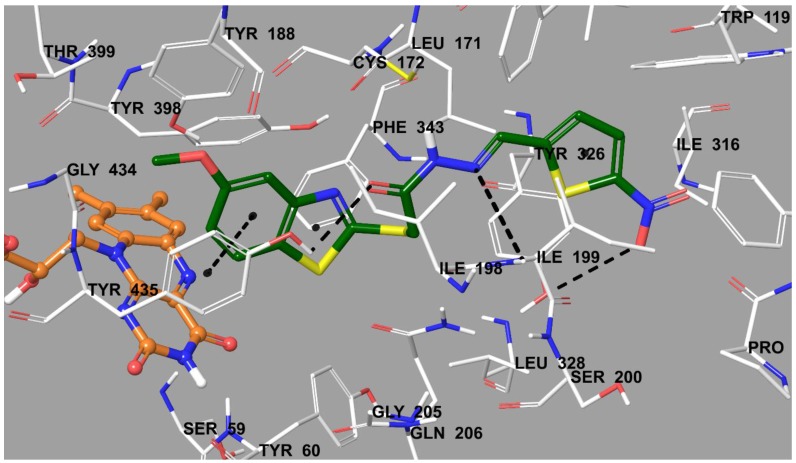
The interacting mode of compound **3e** in the active region of *h*MAO-B. The inhibitor and the important residues in the active site of the enzyme are presented as a tube model. The FAD molecule is colored orange as ball and stick models.

According to enzyme activity assay, it was found that compound **3e** was the most active and selective derivative in the series on hMAO-B enzyme. Its binding modes were obtained with the help of docking studies. The X-ray crystal structure of hMAO-B (PDB ID: 2V5Z) [1] was obtained from Protein Data Bank server [28]. The docking pose of compound **3e** on *h*MAO-B is presented in Figure 3. 

In Figure 3**,** it is seen that the benzothiazole substructure is very near the FAD cofactor, whereas the 5-nitrothiophen substructure has a position in the entrance of the cavity. The benzothiazole moiety creates a π-π interaction with the phenyl of Tyr435. The carbonyl of hydrazone is in an interaction with hydroxyl of Tyr435 by forming a hydrogen bond. The imine nitrogen of hydrazone moiety establishes a hydrogen bond with amino of Ile199. The last interaction is observed between the nitro group and Tyr326. The hydroxyl of this amino acid residue forms a hydrogen bond with the oxygen of the nitro group at the fifth position of thiophene. It is thought that this additional interaction is important for compound **3e** in terms of explaining its inhibitory activity. It may be declared that the presence of an electron withdrawing group such as nitro at this position has a positive contribution to the activity. This suggestion is also supported by compound **3h,** which has a nitro group at the same position of furan ring, and which is the second most active derivative. Prompted from the results of enzyme inhibition and docking studies, it can be suggested that he presence of an electron-withdrawing group, such as the nitro at the fifth position of thiophene or the furan ring, is very important for enzyme inhibitory activity.

## 3. Materials and Methods 

### 3.1. Chemistry

All chemicals were obtained either from Sigma-Aldrich (Sigma-Aldrich Corp., St. Louis, MO, USA) or Merck (Merck KGaA, Darmstadt, Germany), and used without further chemical purification. Melting points of the compounds were measured by using an automatic melting point determination instrument (MP90, Mettler-Toledo, OH, USA) and were presented as uncorrected. ^1^H and ^13^C-NMR spectra were recorded in DMSO-*d*_6_ by a Bruker digital FT-NMR spectrometer (Bruker Bioscience, MA, USA) at 300 MHz and 75 MHz, respectively. The IR spectra of the compounds were recorded using an IRAffinity-1S Fourier transform IR (FTIR) spectrometer (Shimadzu, Tokyo, Japan). The HRMS studies were performed on an LCMS-IT-TOF system (Shimadzu, Tokyo, Japan). Chemical purities of the compounds were checked by classical TLC applications, which was performed on a silica gel 60 F254 (Merck KGaA, Darmstadt, Germany). 

#### 3.1.1. Synthesis of *O*-Ethyl *S*-(5-Methoxybenzothiazol-2-yl) Carbonothioate (**1**)

A mixture of 5-methoxybenzothiazole-2-thiol (30 mmol, 5.74 g), ethyl 2-chloroacetate (30 mmol, 3.21 mL), and potassium carbonate (30 mmol, 4.14 g) was refluxed in acetone for 10 h. After completion of the reaction, the acetone was evaporated, and the precipitated product was washed with deionised water, dried, and recrystallized from EtOH.

#### 3.1.2. Synthesis of *S*-(5-Methoxybenzothiazol-2-yl) Hydrazinecarbothioate (**2**)

*O*-ethyl *S*-(5-methoxybenzothiazol-2-yl) carbonothioate (**1**) (20 mmol, 4.74 g) and an excess of hydrazine hydrate (10 mL) were stirred in EtOH for 8 h. After completion of the reaction, the precipitated product was filtered, washed with cold EtOH, dried, and recrystallized from EtOH.

#### 3.1.3. General Procedure for the Synthesis of the Target Compounds (**3a**–**3j**)

*S*-(5-methoxybenzothiazol-2-yl) hydrazinecarbothioate (**2**) (1.2 mmol, 300 mg), an appropriate heterocyclic benzaldehyde (1.2 mmol), and a catalytic quantity of acetic acid (0.1 mL) were refluxed in EtOH for 2 h. The mixture was cooled, and the precipitated product was filtered, dried, and recrystallized from EtOH.

*2-((5-Methoxybenzothiazol-2-yl)thio)-N′-(thiophen-2-ylmethylene)acetohydrazide* (**3a**). Yield: 81%, M.P. = 163.7–165.3 °C, FTIR (ATR, cm^−1^): 3076 (N-H), 2960 (C-H), 1666 (C=O), 1392 (C=N), 1139 (C-O), 829, 758. ^1^H-NMR (300 MHz, DMSO-*d*_6_): δ = 3.81 (3H, s, -OCH_3_), 4.58 (2H, s, -CH_2_), 6.99 (1H, dd, *J =* 8.80, 2.55 Hz, BT-H_5_), 7.13 (1H, d, *J =* 3.60 Hz, Thiophene CH), 7.38–7.40 (1H, m, BT-H_4_), 7.45–7.48 (1H, m, Thiophene CH), 7.64–7.66 (1H, m, Thiophene CH), 7.87 (1H, d, *J =* 8.80 Hz, BT-H_6_), 8.23 (1H, s, -CH=N), 11.75 (1H, s, -NH). ^13^C-NMR (75 MHz, DMSO-*d*_6_): δ = 35.43, 55.99, 104.97, 114.22, 122.54, 128.43, 129.17, 131.19, 131.68, 139.52, 142.80, 154.38, 159.15, 163.33, 168.34. HRMS (*m*/*z*): [M + H]^+^ calcd for C_15_H_13_N_3_O_2_S_3_: 364.0243; found: 364.0249.

*2-((5-Methoxybenzo[d]thiazol-2-yl)thio)-N′-((3-methylthiophen-2-yl)methylene)acetohydrazide* (**3b**). Yield: 78%, M.P. = 162.9–164.4 °C. FTIR (ATR, cm^−1^): 3066 (N-H), 2949 (C-H), 1668 (C=O), 1363 (C=N), 1136 (C-O), 790, 713. ^1^H-NMR (300 MHz, DMSO-*d*_6_): δ = 2.31 (3H, s, CH_3_), 3.81 (3H, s, OCH_3_), 4.57 (2H, s, –CH_2_-), 6.95–7.03 (2H, m, BT-H_5_, Thiophene CH), 7.40 (1H, d, *J =* 2.43 Hz, BT-H_4_), 7.55 (1H, d, *J =* 5.04 Hz, Thiophene CH), 7.87 (1H, d, *J =* 8.79 Hz, BT-H_6_), 8.29 (1H, s, -CH=N), 11.69 (1H, s, -NH) ^13^C-NMR (75 MHz, DMSO-*d*_6_): δ = 14.13, 35.45, 56.00, 105.01, 114.23, 122.53, 128.20, 131.37, 131.50, 132.43, 138.70, 140.15, 141.90, 159.17, 163.10, 168.13. HRMS (*m*/*z*): [M + H]^+^ calcd for C_16_H_15_N_3_O_2_S_3_: 377.0399; found: 377.0392.

*2-((5-Methoxybenzothiazol-2-yl)thio)-N′-((5-methylthiophen-2-yl)methylene)acetohydrazide* (**3c**). Yield: 75%, M.P. = 149.6–152.3 °C, FTIR (ATR, cm^−1^): 3066 (N-H), 2912 (C-H), 1660 (C=O), 1317 (C=N), 1199 (C-O), 794, 717. ^1^H-NMR (300 MHz, DMSO-*d*_6_): δ = 2.44 (3H, s, CH_3_), 3.82 (3H, s, OCH_3_), 4.55 (2H, s, –CH_2_-), 6.81–6.83 (1H, m, Thiophene CH), 6.99 (1H, dd, *J =* 8.80, 2.52 Hz, BT-H_5_), 7.24 (1H, d, *J =* 3.60 Hz, Thiophene CH), 7.41 (1H, d, *J =* 2.46 Hz, BT-H_4_), 7.87 (1H, d, *J =* 8.79 Hz, BT-H_6_), 8.13 (1H, s, -CH=N), 11.69 (1H, s, -NH). ^13^C-NMR (75 MHz, DMSO-*d*_6_): δ = 15.74, 35.38, 56.01, 105.03, 114.22, 122.53, 126.80, 131.51, 132.09, 136.95, 139.70, 143.07, 154.42, 159.17, 163.17, 168.26. HRMS (*m*/*z*): [M + H]^+^ calcd for C_16_H_15_N_3_O_2_S_3_: 378.0399; found: 378.0390.

*N′-((5-Bromothiophen-2-yl)methylene)-2-((5-methoxybenzothiazol-2-yl)thio)acetohydrazide* (**3d**). Yield: 84%, M.P. = 188.3–190.4 °C, FTIR (ATR, cm^−1^): 3078 (N-H), 2958 (C-H), 1672 (C=O), 1359 (C=N), 1193 (C-O), 786, 758. ^1^H-NMR (300 MHz, DMSO-*d*_6_): δ 3.82 (3H, s, OCH_3_), 4.55 (2H, s, –CH_2_-), 6.99 (1H, dd, *J =* 8.82, 2.52 Hz, BT-H_5_), 7.24–7.27 (1H, m, Thiophene CH), 7.29–7.33 (1H, m, Thiophene CH), 7.40 (1H, d, *J =* 2.46 Hz, BT-H_4_), 7.87 (1H, d, *J =* 8.82 Hz, BT-H_6_), 8.15 (1H, s, -CH=N), 11.86 (1H, s, NH) ^13^C-NMR (75 MHz, DMSO-*d*_6_): δ = 35.23, 56.00, 105.01, 114.28, 122.55, 131.69, 131.79, 132.20, 138.62, 141.09, 142.06, 154.41, 159.18, 163.46, 168.50. HRMS (*m*/*z*): [M + H]^+^ calcd for C_15_H_12_N_3_O_2_S_3_Br: 441.9348; found: 441.9343.

*2-((5-Methoxybenzothiazol-2-yl)thio)-N′-((5-nitrothiophen-2-yl)methylene)acetohydrazide* (**3e**). Yield: 92%, M.P. = 177.2–178.6 °C, FTIR (ATR, cm^−1^): 3095 (N-H), 2962 (C-H), 1666 (C=O), 1359 (C=N), 1193 (C-O), 794, 732. ^1^H-NMR (300 MHz, DMSO-*d*_6_): δ 3.81 (3H, s, -OCH_3_), 4.62 (2H, s, -CH_2_), 6.99 (1H, dd, *J =* 8.79, 2.52 Hz, BT-H_5_), 7.39 (1H, d, *J =* 2.55 Hz, BT-H_4_), 7.55–7.58 (1H, m, Thiophene CH), 7.87 (1H, d, *J =* 8.79 Hz, BT-H_6_), 8.11 (1H, d, *J =* 4.32 Hz, Thiophene CH), 8.24 (1H, s, -CH=N), 12.14 (1H, s, NH) ^13^C-NMR (75 MHz, DMSO-*d*_6_): δ = 35.15, 55.98, 105.01, 114.28, 122.57, 126.78, 129.91, 131.01, 137.71, 141.22, 146.62, 154.33, 159.18, 164.09, 169.07. HRMS (*m*/*z*): [M + H]^+^ calcd for C_15_H_12_N_4_O_4_S_3_: 409.0093; found: 409.0087.

*N′-(Furan-2-ylmethylene)-2-((5-methoxybenzothiazol-2-yl)thio)acetohydrazide* (**3f**). Yield: 79%, M.P. = 146.1–147.9 °C, FTIR (ATR, cm^−1^): 3136 (N-H), 2949 (C-H), 1654 (C=O), 1386 (C=N), 1193 (C-O), 758, 696. ^1^H-NMR (300 MHz, DMSO-*d*_6_): δ 3.82 (3H, s, -OCH_3_), 4.62 (2H, s, -CH_2_), 6.61–6.64 (1H, m, Furan CH), 6.92 (1H, d, *J =* 3.42 Hz, Furan CH), 6.99 (1H, dd, *J =* 8.82, 2.49 Hz, BT-H_5_), 7.41 (1H, d, *J =* 2.46 Hz, BT-H_4_), 7.82–7.83 (1H, m, Furan CH), 7.86 (1H, d, *J =* 8.82 Hz, BT-H_6_), 7.95 (1H, s, -CH=N), 11.70 (1H, s, -NH), ^13^C-NMR (75 MHz, DMSO-*d*_6_): δ = 35.82, 55.99, 104.98, 112.65, 114.43, 122.52, 126.67 134.55, 137.45, 145.61, 149.40, 154.39, 159.15, 163.44, 168.44,. HRMS (*m*/*z*): [M + H]^+^ calcd for C_15_H_13_N_3_O_3_S_2_: 348.0471; found: 348.0466.

*2-((5-Methoxybenzothiazol-2-yl)thio)-N′-((5-methylfuran-2-yl)methylene)acetohydrazide* (**3g**). Yield: 79%, M.P. = 145.3–146.7 °C, FTIR (ATR, cm^−1^): 3061 (N-H), 2962 (C-H), 1658 (C=O), 1377 (C=N), 1193 (C-O), 785, 694. ^1^H-NMR (300 MHz, DMSO-*d*_6_): δ 2.34 (3H, s, -CH_3_) 3.82 (3H, s, -OCH_3_), 4.61 (2H, s, -CH_2_-), 6.25–6.26 (1H, m, Furan CH), 6.80 (1H d, *J =* 3.24 Hz, Furan CH), 6.99 (1H, dd, *J =* 8.79, 2.52 Hz, BT-H_5_), 7.42 (1H, d, *J =* 2.43 Hz, BT-H_4_), 7.86 (1H, d, *J =* 8.73 Hz, BT-H_6_), 7.87 (1H, s, -CH=N), 11.60 (1H, s, -NH). ^13^C-NMR (75 MHz, DMSO-*d*_6_): δ = 14.01, 35.86, 55.99, 105.00, 109.07, 114.19, 116.15, 122.51, 126.68, 134.68, 137.33, 147.92, 155.10, 159.15, 163.27, 168.23. HRMS (*m*/*z*): [M + H]^+^ calcd for C_16_H_15_N_3_O_3_S_2_: 362.0628; found: 362.0632.

*2-((5-Methoxybenzothiazol-2-yl)thio)-N′-((5-nitrofuran-2-yl)methylene)acetohydrazide* (**3h**). Yield: 80%, M.P. = 168.9–170.7 °C, FTIR (ATR, cm^−1^): 3211 (N-H), 2999 (C-H), 1674 (C=O), 1309 (C=N), 1197 (C-O), 790, 738. ^1^H-NMR (300 MHz, DMSO-*d*_6_): δ 3.81 (3H, s, -OCH_3_), 4.67 (2H, s, -CH_2_), 6.99 (1H, dd, *J =* 8.82, 2.49 Hz, BT-H_5_), 7.28 (1H, d, *J =* 3.96 Hz, Furan CH), 7.38–7.39 (1H, m, BT-H_4_), 7.79 (1H, d, *J =* 3.96 Hz, Furan CH), 7.86 (1H, d, *J =* 8.79 Hz, BT-H_6_), 8.06 (1H, s, -CH=N), 12.20 (1H, s, -NH), ^13^C-NMR (75 MHz, DMSO-*d*_6_): δ = 35.50, 56.00, 105.02, 114.25, 115.16, 115.60, 122.54, 126.75, 132.58, 135.53, 151.82, 154.31, 159.16, 164.20, 169.05. HRMS (*m*/*z*): [M + H]^+^ calcd for C_15_H_12_N_4_O_5_S_2_: 393.0322; found: 393.0315.

*2-((5-Methoxybenzothiazol-2-yl)thio)-N′-(pyridin-3-ylmethylene)acetohydrazide* (**3i**). Yield: 82%, M.P. = 177.5–179.3 °C, FTIR (ATR, cm^−1^): 3192 (N-H), 2927 (C-H), 2341, 1699 (C=O), 1309 (C=N), 1186 (C-O), 702, 692. ^1^H-NMR (300 MHz, DMSO-*d*_6_): δ 3.81 (3H, s, -OCH_3_), 4.69 (2H, s, -CH_2_), 6.99 (1H, dd, *J =* 8.79, 2.52 Hz, BT-H_5_), 7.39 (1H, d, *J =* 2.43 Hz, BT-H_4_), 7.43–7.47 (1H, m, Pyridine CH), 7.86 (1H, d, *J =* 8.79 Hz, BT-H_6_), 8.10–8.14 (2H, m, Pyridine CH, -CH=N), 8.60 (1H, dd, *J =* 4.77, 1.50 Hz, Pyridine CH), 8.88 (1H, d, *J =* 1.56 Hz, Pyridine CH), 11.92 (1H, s, NH), ^13^C-NMR (75 MHz, DMSO-*d*_6_): δ = 35.50, 55.98, 104.97, 114.25, 122.54, 124.39, 126.73, 130.37, 133.94, 141.56, 149.02, 151.06, 154,36, 159.15, 163.72, 168.97. HRMS (*m*/*z*): [M + H]^+^ calcd for C_16_H_14_N_4_O_2_S_2_: 359.0631; found: 359.0635.

*2-((5-Methoxybenzothiazol-2-yl)thio)-N′-(pyridin-4-ylmethylene)acetohydrazide* (**3j**). Yield: 82%, M.P. = 159.8–161.4 °C, FTIR (ATR, cm^−1^): 3086 (N-H), 2922 (C-H), 2358, 1693 (C=O), 1307 (C=N), 1192 (C-O), 783, 759. ^1^H-NMR (300 MHz, DMSO-*d*_6_): δ 3.81 (3H, s, -OCH_3_), 4.70 (2H, s, -CH_2_-), 6.99 (1H, dd, *J =* 8.79, 2.52 Hz, BT-H_5_), 7.39 (1H, d, *J =* 2.52 Hz, BT-H_4_), 7.65 (2H, d, *J =* 6.00 Hz, Pyridine CH), 7.86 (1H, d, *J =* 8.79 Hz, BT-H_6_), 8.05 (1H, s, -CH=N), 8.62 (2H, d, *J =* 6.06 Hz, Pyridine CH), 12.04 (1H, s, -NH). ^13^C-NMR (75 MHz, DMSO-*d*_6_): δ = 35.41, 55.98, 104.99, 114.26, 121.32, 122.56, 126.76, 141.90, 145.26, 150.71, 154.34, 159.16, 164.02, 169.23. HRMS (*m*/*z*): [M + H]^+^ calcd for C_16_H_14_N_4_O_2_S_2_: 359.0631; found: 359.0628.

### 3.2. Activity Studies

#### 3.2.1. MAO-A and MAO-B Inhibition Assay

An enzyme inhibition assay was performed according to our recent study [21]. The inhibitor (20 μL/well) and *h*MAO-A (100 μL/well) or *h*MAO-B solution (100 μL/well) was added to the fine black-bottom 96-well microtiter plate and the mixture was incubated at 37 °C for 30 min. After the incubation period, the reaction was started by adding a working solution (100 μL/well) (200 mM Amplex Red reagent, 1 U/mL horseradish peroxidase and 1 mM p-tyramine) and all pipetting processes were performed using a Biotek Precision XS robotic system (BioTek Instruments, Winooski, VT, USA).

The production of H_2_O_2_ and, subsequently, of resorufin was quantified at 37 °C in a BioTek-Synergy H1 microplate reader based on the fluorescence generated (excitation, 535 nm, emission, 587 nm) over a 15 min period, in which the fluorescence increased linearly. Control experiments were carried out simultaneously by replacing the inhibitor solution with 2% DMSO (20 μL). In order to check the probable inhibitory effect of inhibitors on horseradish peroxidase, a parallel reading was achieved by replacing enzyme solutions with 3% H_2_O_2_ solution (20 mM 100 μL/well). In addition, the probable capacity of the inhibitors to modify the fluorescence generated in the reaction mixture due to non-enzymatic inhibition was determined by mixing inhibitor and working solutions.

The specific fluorescence emission (used to obtain the final results) was calculated after subtraction of the background activity, which was determined from vials containing all components except the *h*MAO isoforms, which were replaced by phosphate buffer (100 μL/well). Blank, control and all concentrations of inhibitors were analyzed in quadruplicate and inhibition percent was calculated by using following equation:

$$\%\;\mathrm{Inhibition}=\frac{\left({\mathrm{FC}}_{t2}-{\mathrm{FC}}_{t1}\right)-\left({\mathrm{FI}}_{t2}-{\mathrm{FI}}_{t1}\right)}{{\mathrm{FC}}_{t2}-{\mathrm{FC}}_{t1}}\times 100$$

where FC_t2_ is the fluorescence of a control well measured at t_2_ time; FC_t1_ is the fluorescence of a control well measured at t_1_ time; FI_t2_ is the fluorescence of an inhibitor well measured at t_2_ time; and FI_t1_ is the fluorescence of an inhibitor well measured at t_1_ time.

The IC_50_ values were calculated from a dose-response curve obtained by plotting the percentage inhibition versus the log concentration with the use of GraphPad PRISM software (version 5.0, GraphPad Software Inc., La Jolla, CA, USA). The results are displayed as the mean ± standard deviation (SD).

#### 3.2.2. Enzyme Kinetic Studies 

The same materials were used in the MAO inhibition assay. The most active compound, **3e**, was studied at three different concentrations (IC_50_/2, IC_50_, and 2 × IC_50_). The solutions of inhibitor (20 μL/well) and enzyme added to the fine black-bottom 96-well microtiter plate and then the mixture was incubated at 37 °C for 30 min. After incubation period, the working solution, including various concentrations (20, 10, 5, 2.5, 1.25, and 0.625 μM) of tyramine (100 μL/well), was added. The increase of the fluorescence (Ex/Em = 535/587 nm) was recorded for 30 min. A parallel experiment was performed without using an inhibitor. All processes were assayed in quadruplicate. Lineweaver-Burk plots were obtained by using Microsoft Office Excel 2013 to analyze the inhibition type. The slopes of the Lineweaver-Burk plots were replotted versus the inhibitor concentration, and the K_i_ values were determined from the x-axis intercept as −K_i_ [21].

### 3.3. Cytotoxicity Test

NIH/3T3 mouse embryonic fibroblast cell line (ATCC^®^ CRL-1658™, London, UK) was used in the cytotoxicity test and, initially, NIH/3T3 cells were incubated in Dulbecco’s Modified Eagle’s Medium (DMEM). NIH/3T3 cells were added to 96-well culture plates (10,000 cells/well). Synthesized compounds and reference agents were added to the 96-well culture plates at various concentrations ranging from 1000 µM to 0.316 µM. MTT assay was performed as previously described [29,30,31]. Dose-response curves were plotted against compound concentrations applied to determine IC_50_ values. The following formula was used to calculate the inhibition percentage for each concentration:

% inhibition = 100 − (mean sample × 100/mean solvent)

### 3.4. Prediction of ADME Parameters and BBB Permeability

Online Molinspiration property calculation program was used to predict ADME parameters of compounds (**3a**–**3j**) [25]. The BBB permeability of the compounds was evaluated by an online BBB predictor [27]. 

### 3.5. Molecular Docking Studies

A structure based in silico procedure was applied to discover the binding modes of compound **3e** to *h*MAO-B enzyme active sites. The crystal structure *h*MAO-B (PDB ID: 1S2Q) [1], which was crystallized with safinamide, were retrieved from the Protein Data Bank server (www.pdb.org). 

The structures of ligands were built using the Schrödinger Maestro [32] interface and then were submitted to the Protein Preparation Wizard protocol of the Schrödinger Suite 2016 Update 2 [33]. The ligands were prepared by the LigPrep 3.8 [34] to assign the protonation states at pH 7.4 ± 1.0 and the atom types, correctly. Bond orders were assigned and hydrogen atoms were added to the structures. The grid generation was formed using Glide 7.1 [35]. The grid box with dimensions of 20 Å × 20 Å × 20 Å was centered in the vicinity of the flavin (FAD) N5 atom on the catalytic site of the protein to cover all binding sites and neighboring residues [36,37,38]. Flexible docking runs were performed with single precision docking mode (SP). 

## 4. Conclusions

Synthesis of novel compounds, incorporating significant pharmacophore groups, is an important medicinal chemistry principle to develop new biologically-active agents. Depending on this approach, in this study, 10 new benzothiazole compounds carrying hydrazone and heteroaryl groups were designed and synthesized as selective *h*MAO-B inhibitors. Enzymatic studies confirmed the selectivity of compounds toward *h*MAO-B enzyme and displayed the potency of compound **3e** against this enzyme. Toxicological and ADME studies enhanced the biological importance of this compound. The docking studies clearly explained the molecular interactions between the compound **3e** and *h*MAO-B. In conclusion, this information may have an effect on medicinal chemists to synthesize more potent and safer *h*MAO-B inhibitors, which may be helpful for the treatment of patients with PD.

## Figures and Tables

**Table:** 

Compounds	Het
**3a**	Thiophen-2yl
**3b**	3-Methylthiophen-2yl
**3c**	5-Methylthiophen-2yl
**3d**	5-Bromothiophen-2yl
**3e**	5-Nitrothiophen-2yl
**3f**	Furan-2yl
**3g**	5-Methylfuran-2yl
**3h**	5-Nitrofuran-2yl
**3i**	Pyridine-3yl
**3j**	Pyridine-4yl

**Table 1:** Percent inhibition of compounds **3a**–**3j**, moclobemide and selegiline against MAO-A and MAO-B at 10^−3^ M to 10^−4^ M concentrations.

Comp.	MAO A Inhibition %		MAO B Inhibition %	
	10^−3^ M	10^−4^ M	10^−3^ M	10^−4^ M
**3a**	25.00	19.02	60.37	52.15
**3b**	30.08	20.45	30.46	26.88
**3c**	14.10	10.75	25.75	18.20
**3d**	18.88	12.71	23.97	17.75
**3e**	22.12	18.55	87.28	83.50
**3f**	30.77	18.30	75.66	68.30
**3g**	16.69	11.07	18.20	11.23
**3h**	25.89	21.30	82.58	79.10
**3i**	19.88	10.55	32.02	28.75
**3j**	22.15	17.28	34.60	30.50
**Moclobemide**	91.42 ± 4.60	77.86 ± 3.71	-	-
**Selegiline**	-	-	97.69 ± 4.16	94.42 ± 3.89

**Table 2:** IC_50_ values of **3a**, **3e, 3f, and 3h** and selegiline against MAO-B.

Comp.	MAO B IC_50_ (µM)
**3a**	15.450 ± 0.398
**3e**	0.060 ± 0.002
**3f**	0.963 ± 0.033
**3h**	0.075 ± 0.003
**Selegiline**	0.044 ± 0.002

**Table 3:** Cytotoxic activity of the compound **3e** against NIH/3T3 cell line.

Comp.	IC_50_ (µM)
**3e**	19.002 ± 1.029

**Table 4:** Some physicochemical parameters of the compounds **3a**–**3j** and reference drugs used in the prediction of ADME profiles.

Comp.	MW (g/mol)	logP	TPSA (ANG^2^)	HBA	HBD	Vol (ANG^3^)	Vio	BBB
**3a**	363.49	4.10	63.59	6	1	291.83	0	+
**3b**	377.52	4.47	63.59	6	1	308.39	0	+
**3c**	377.52	4.32	63.59	6	1	308.39	0	+
**3d**	442.38	5.03	63.59	6	1	309.72	1	+
**3e**	408.49	4.18	109.41	7	1	315.17	0	+
**3f**	347.42	3.46	76.73	6	1	282.69	0	+
**3g**	361.45	3.68	76.73	6	1	299.25	0	+
**3h**	392.42	3.54	122.55	7	1	306.02	0	+
**3i**	358.45	2.96	76.48	6	1	296.96	0	+
**3j**	358.45	2.91	76.48	6	1	296.96	0	+
**Selegiline**	187.29	2.64	3.24	1	0	202.64	0	+
**Clorgiline**	272.18	3.74	12.47	2	0	238.91	0	+

## References

[1] Binda C., Wang J., Pisani L., Caccia C., Carotti A., Salvati P., Edmondson D., Mattevi A. (2007). Structures of Human Monoamine Oxidase B Complexes with Selective Noncovalent Inhibitors:  Safinamide and Coumarin Analogs. J. Med. Chem..

[2] Choi J.W., Jang B.K., Cho N.C., Park J.H., Yeon S.K., Ju E.J., Lee Y.S., Han G., Pae A.N., Kim D.J. (2015). Synthesis of a series of unsaturated ketone derivatives as selective and reversible monoamine oxidase inhibitors. Bioorg. Med. Chem..

[3] Finberg J.P.M., Rabey J.M. (2016). Inhibitors of MAO-A and MAO-B in Psychiatry and Neurology. Front. Pharmacol..

[4] Livingston M.G., Livingston H.M. (1996). Monoamine oxidase inhibitors. An update on drug interactions. Drug Saf..

[5] Fiedorowicz J.G., Swartz K.L. (2004). The Role of Monoamine Oxidase Inhibitors in Current Psychiatric Practice. J. Psychiatr. Pract..

[6] Riederer P., Laux G. (2011). MAO-inhibitors in Parkinson’s Disease. Exp. Neurobiol..

[7] Alexander G.E. (2004). Biology of Parkinson’s disease: Pathogenesis and pathophysiology of a multisystem neurodegenerative disorder. Dialogues Clin. Neurosci..

[8] Dickson D.W. (2012). Parkinson’s Disease and Parkinsonism: Neuropathology. Cold Spring Harb. Perspect. Med..

[9] Goldenberg M.M. (2008). Medical Management of Parkinson’s Disease. Pharm. Ther..

[10] Dezsi L., Vecsei L. (2017). Monoamine Oxidase B Inhibitors in Parkinson’s Disease. CNS Neurol. Disord. Drug Targets.

[11] Teo K.C., Ho S.L. (2013). Monoamine oxidase-B (MAO-B) inhibitors: Implications for disease-modification in Parkinson’s disease. Transl. Neurodegener..

[12] Robakis D., Fahn S. (2015). Defining the Role of the Monoamine Oxidase-B Inhibitors for Parkinson’s Disease. CNS Drugs.

[13] Wang Z., Wu J., Yang X., Cai P., Liu Q., Wang K.D.G., Kong L., Wang X. (2016). Neuroprotective effects of benzyloxy substituted small molecule monoamine oxidase B inhibitors in Parkinson’s disease. Bioorg. Med. Chem..

[14] Binda C., Newton-Vinson P., Hubalek F., Edmondson D.E., Mattevi A. (2002). Structure of human monoamine oxidase B, a drug target for the treatment of neurological disorders. Nat. Struct. Biol..

[15] Binda C., Newton-Vinson P., Hubalek F., Edmondson D.E., Mattevi A. (2004). Crystal structure of human monoamine oxidase B, a drug target enzyme monotopically inserted into the mitochondrial outer membrane. FEBS Lett..

[16] Tripathi R.K., Goshain O., Ayyannan S.R. (2013). Design, synthesis, in vitro MAO-B inhibitory evaluation, and computational studies of some 6-nitrobenzothiazole-derived semicarbazones. ChemMedChem.

[17] Kaya B., Sağlık B.N., Levent S., Özkay Y., Kaplancıklı Z.A. (2016). Synthesis of some novel 2-substituted benzothiazole derivatives containing benzylamine moiety as monoamine oxidase inhibitory agents. J. Enzyme Inhib. Med. Chem..

[18] Tripathi R.K., Ayyannan S.R. (2016). Design, Synthesis, and Evaluation of 2-Amino-6-nitrobenzothiazole-Derived Hydrazones as MAO Inhibitors: Role of the Methylene Spacer Group. ChemMedChem.

[19] Lammana C., Sinicropi M.S., Pietrangeli P., Corbo F., Franchini C., Mondovi B., Perrone M.G., Scilimati A. (2004). Synthesis and biological evaluation of 3-alkyloxazolidine-2-ones as reversible MAO inhibitors. Arkivoc.

[20] Chimenti P., Petzer A., Carradori S., D’Ascenzio M., Silvestri R., Alcaro S., Ortuso F., Petzer J.P., Secci D. (2013). Exploring 4-substituted-2-thiazolylhydrazones from 2-, 3-, and 4-acetylpyridine as selective and reversible hMAO-B inhibitors. Eur. J. Med. Chem..

[21] Can Ö.D., Osmaniye D., Demir Özkay Ü., Sağlık B.N., Levent S., Ilgın S., Baysal M., Özkay Y., Kaplancıklı Z.A. (2017). MAO enzymes inhibitory activity of new benzimidazole derivatives including hydrazone and propargyl side chains. Eur. J. Med. Chem..

[22] Can N.Ö., Osmaniye D., Levent S., Sağlık B.N., İnci B., Ilgın S., Özkay Y., Kaplancıklı Z.A. (2017). Synthesis of New Hydrazone Derivatives for MAO Enzymes Inhibitory Activity. Molecules.

[23] International Organization for Standardization (2009). International Organization for Standardization, Biological Evaluation of Medical Devices-Part 5: Tests for in Vitro Cytotoxicity ISO-10993-5.

[24] De Waterbeemd H.V., Gifford E. (2013). ADMET in silico modelling: Towards prediction paradise?. Nat. Rev. Drug Discov..

[25] Molinspiration Cheminformatics, Bratislava, Slovak Republic. http://www.molinspiration.com/services/properties.html.

[26] Carpenter T.S., Kirshner D.A., Lau E.Y., Wong S.E., Nilmeier J.P., Lightstone F.C. (2014). A Method to Predict Blood-Brain Barrier Permeability of Drug-Like Compounds Using Molecular Dynamics Simulations. Biophys. J..

[27] About the Blood-brain Barrier (BBB) Prediction Server. http://www.cbligand.org/BBB/index.php.

[28] Protein Data Bank Server. http://www.pdb.org.

[29] Karaca Gençer H., Acar Çevik U., Kaya Çavuşoğlu B., Sağlık B.N., Levent S., Atlı Ö., Ilgın S., Özkay Y., Kaplancıklı Z.A. (2017). Design, synthesis, and evaluation of novel 2-phenylpropionic acid derivatives as dual COX inhibitory-antibacterial agents. J. Enzyme Inhib. Med. Chem..

[30] Demir Özkay Ü., Can Ö.D., Sağlık B.N., Acar Çevik U., Levent S., Özkay Y., Ilgın S., Atlı Ö. (2016). Design, synthesis, and AChE inhibitory activity of new benzothiazole-piperazines. Bioorg. Med. Chem. Lett..

[31] Karaca Gençer H., Levent S., Acar Çevik U., Özkay Y., Ilgın S. (2016). New 1,4-dihydro[1,8]naphthyridine derivatives as DNA gyrase inhibitors. Bioorg. Med. Chem. Lett..

[32] (2016). Maestro, Version 10.6.

[33] (2016). Schrödinger, Version 2016-2.

[34] (2016). LigPrep, Version 3.8.

[35] (2016). Glide, Version 7.1.

[36] Toprakçı M., Yelekçi K. (2005). Docking studies on monoamine oxidase-B inhibitors: Estimation of inhibition constants (Ki) of a series of experimentally tested compounds. Bioorg. Med. Chem. Lett..

[37] Gökhan-Kelekçi N., Özgün Şimşek Ö., Ercan A., Yelekçi K., Sibel Şahin Z., Işık Ş., Uçar G., Bilgin A.A. (2009). Synthesis and molecular modeling of some novel hexahydroindazole derivatives as potent monoamine oxidase inhibitors. Bioorg. Med. Chem..

[38] Evranos-Aksöz B., Yabanoğlu-Çiftçi S., Uçar G., Yelekçi K., Ertan R. (2014). Monoamine oxidase inhibitory activities and docking studies. Bioorg. Med. Chem. Lett..

